# Effect of Additional Iron Injection to Suckling Pigs on Hematocrit Level during the Suckling Period

**DOI:** 10.3390/ani12212980

**Published:** 2022-10-30

**Authors:** Sarah E. Albers, Emily A. Pintens, Paige K. Isensee, Clara M. Lemanski, Young Dal Jang

**Affiliations:** Department of Animal and Food Science, University of Wisconsin-River Falls, River Falls, WI 54022, USA

**Keywords:** iron, additional injection, blood, hematocrit, suckling piglets

## Abstract

**Simple Summary:**

Piglets are susceptible to iron-deficiency anemia since they are born with low iron reserve and sow milk contains low content of iron. A single iron injection is a common practice in swine industry but may not be sufficient to meet iron requirement during the entire suckling period when pigs are weaned at the age over 21 d. A second iron injection in the suckling period could be a useful way to boost iron status and thereby help to improve oxygen carrying capacity of suckling pigs. However, it has not been demonstrated yet if there is a difference in the effectiveness of second iron injection to piglets weaned at a longer age (approximately 28 d of age) when injected at different doses and when having different hematocrit status at the time of administration. The current study aims at demonstrating the effect of an additional iron injection on hematocrit levels in suckling pigs when different levels of iron were administered to pigs at birth or in the suckling period. The result of current study suggested that the additional iron injection to suckling pigs could increase hematocrit levels. The amount of iron in the first (at birth) and second (before weaning) injections could affect the effectiveness of the second iron injection on blood hematocrit level.

**Abstract:**

Two experiments were conducted to evaluate the effects of additional iron injection to suckling piglets on hematocrit level during the suckling period. In Experiment 1, a total of 24 piglets were allotted into 3 treatments within litter based on body weight and sex at d 1 of age. Treatments were: (1) Control: a 200 mg iron-dextran intramuscular injection only at d 0 of experiment (d 1 of age), (2) Iron100: intramuscular iron-dextran injections at d 0 (200 mg iron) and 15 (11 d before weaning) of experiment (100 mg iron), and (3) Iron200: intramuscular iron-dextran injections at d 0 (200 mg iron) and 15 of experiment (200 mg iron). In Experiment 2, a total of 20 piglets were allotted into 2 treatments within litter based on body weight and sex at d 1 to 2 of age. Treatments were: (1) Iron100b: 100 mg iron-dextran intramuscular injection at d 0 of experiment (d 1 to 2 of age), and (2) Iron200b: 200 mg iron-dextran intramuscular injection at d 0 of experiment. An additional 200 mg iron-dextran was injected to all piglets intramuscularly at d 14 of experiment (11 d before weaning). In Experiment 1, there was no difference in hematocrit levels among treatments at d 15 of experiment. Both iron treatments had greater hematocrit levels than the Control treatment at d 22 and 26 of experiment (*p* < 0.05). The Iron200 treatment had greater hematocrit level at d 26 of experiment (*p* < 0.05) and tended to have a greater increase of hematocrit levels in d 22 to 26 of experiment (*p* = 0.09) than the Iron100 treatment. In Experiment 2, the Iron200b treatment had greater hematocrit levels than the Iron100b treatment in d 14 and 25 (*p* < 0.05) of experiment. Hematocrit level changes tended to be greater in the Iron200b treatment in d 0 to 14 (*p* = 0.08) of experiment but lower in d 14 to 25 (*p* < 0.05) of experiment than the Iron100b treatment. The additional iron injection to suckling piglets increased hematocrit levels with greater values at weaning in the higher injection level whereas the increase was greater when the hematocrit level was low at the time of additional injection.

## 1. Introduction

Newborn piglets are susceptible to iron deficiency as they are born with insufficient iron reserve at 50 mg and sow milk contains low level of iron at 1 mg/L although the piglets require 7–16 mg of iron per day [1,2]. Iron injection at birth with 150–200 mg is a common practice to prevent piglets from iron deficiency anemia during the suckling period in pig production [1]. However, Friendship et al. [3] reported that a single iron injection at d 4 of age was not sufficient to prevent iron deficiency at weaning ranged from 16 to 21 d. Perri et al. [4] also reported that the traditional 200 mg of iron injection is insufficient to meet the needs for iron for the large and fast-growing piglets. As piglets grow fast during the suckling period and weaning weight increases with increasing weaning age up to d 28 of age, a single iron injection at birth may not be sufficient to prevent large piglets from anemia at weaning, which may result in growth retardation afterward [5,6,7]. However, Williams et al. [8] reported that intramuscular injection of a 200 mg iron within 7 d after birth was sufficient to optimize pre- and postweaning growth performance of pigs. Friendship et al. [3] reported that daily access to iron-enriched peat moss or a 200 mg of second iron injection intramuscularly at d 14 of age could be effective in meeting iron requirement of suckling piglets. In those studies, the piglets were weaned at 21 d after birth and it could not demonstrate the effectiveness of a second iron injection when the weaning age was around 26–28 d after birth. In addition, Yu et al. [9] suggested that creep feeding with 175 mg Fe/kg diet from d 7 of age might contribute the certain amount of iron to the piglets for sufficient overall growth while there was a numerically lower growth rate, significantly lower serum iron and hemoglobin levels in no additional iron injection treatment compared with the additional iron injection treatments. Due to low absorption rate and less tissue retention of iron administered orally [10,11], an additional iron injection before weaning might be needed to improve hematological status and potentially growth of piglets [12,13,14] when the pigs are weaned at over 26–28 d of age although an additional iron injection could be laborious in the pig production [15]. Furthermore, it has not been demonstrated yet whether the effectiveness of additional iron injection depends on the hematological status of piglets at the administration that are dependent on the level of the first iron injection at birth [14] and the amount of iron additionally administered to piglets prior to weaning [6]. Therefore, the objective of the current study was to evaluate the effects of additional iron injection in suckling piglets when administered before weaning or with two levels of iron at birth on hematocrit levels during the suckling period as the hematocrit is a representative indicator of hematological status of pigs that is positively related to hemoglobin level and potential growth of piglets after weaning [16].

## 2. Materials and Methods

### 2.1. Animal Care

All procedures used in the experiments were approved by the Institutional Animal Care and Use Committee of University of Wisconsin-River Falls (Protocol #19-20-8 and 19-20-13 for Experiment 1 and 2, respectively). The experiments were conducted in the farrowing facility at Mann Valley Farm of University of Wisconsin-River Falls (WI, USA).

### 2.2. Animals and Experimental Design

For Experiment 1, a total of 24 suckling piglets (Yorkshire × Yorkshire and Yorkshire × Duroc) from 4 litters (average litter size: 10.25; 9 to 11 piglets; average 3.5 parities) at d 1 of age were allotted into 3 treatments within litter (8 pigs per treatment; 4 barrows and 4 gilts) based on body weight and sex as follows: 1) Control: a 200 mg iron-dextran (200 mg iron/mL; Uniferon^®^ 200, Pharmacosmos, Inc., Watchung, NJ, USA) intramuscular injection only at d 0 of experiment (d 1 of age), 2) Iron100: intramuscular iron-dextran injections at d 0 (200 mg iron) and 15 of experiment (100 mg iron), and 3) Iron200: intramuscular iron-dextran injections at d 0 (200 mg iron) and 15 of experiment (200 mg iron). The initial 200 mg iron-dextran was injected intramuscularly on d 0 of experiment (d 1 of age) with an additional injection given to the piglets at d 15 of experiment (11 d before weaning). Body weight and hematocrit level were measured at d 0 (body weight only), 15, 22, and 26 of experiment. All piglets were weaned at d 26 of experiment (d 27 of age). No creep feed was offered to piglets throughout the suckling period.

For Experiment 2, a total of 20 suckling piglets (Yorkshire × Yorkshire and Yorkshire × Duroc) from 5 litters (average litter size: 10.60; 9 to 12 piglets; average 2.4 parities) at d 1 to 2 of age were allotted into 2 treatments within litter (10 pigs per treatment; 5 barrows and 5 gilts) based on body weight and sex as follows: 1) Iron100b: intramuscular injection of 100 mg iron-dextran at d 0 of experiment (d 1 to 2 of age), and 2) Iron200b: intramuscular injection of 200 mg iron-dextran at d 0 of experiment. A 200 mg iron-dextran was injected to all piglets intramuscularly at d 14 of experiment (d 15 to 16 of age; 11 d before weaning). Body weight and hematocrit level were measured at d 0, 7, 14 (before additional injection), 21, and 25 (weaning) of experiment. All piglets were weaned at d 25 of experiment (d 26 to 27 of age). No creep feed was offered to piglets throughout the suckling period.

Each pig was injected with iron in the right neck muscle intramuscularly and a commercial iron-dextran product was used in Experiments 1 and 2. In both experiments, 0.5 and 1.0 mL of the iron-dextran product were injected to piglets for 100 and 200 mg of iron treatments, respectively. 

### 2.3. Housing, Data and Sample Collection, and Analysis

All sows with suckling piglets in Experiments 1 and 2 were housed in raised-deck farrowing crates (1.52 × 2.13 m^2^) with tenderfoot or woven-wire flooring in an environmentally controlled farrowing facility. A typical lactation diet was provided to all sows ad libitum with free access to water from a water nipple throughout the entire experimental period. All piglets were processed at the time of first iron injection. Processing of the piglets involved weighing, ear-notching, tail docking, and castration.

For Experiment 1, blood samples were collected from the jugular vein into a glass tube containing K_3_EDTA at d 15 (before additional iron injection administration), 22, and 26 (weaning) of experiment for determination of hematocrit levels. For hematocrit level determination, two 75 mm sodium heparinized capillary tubes (Jorgensen Laboratories Inc., Loveland, CO, USA) were filled with blood from each blood sample and then packed with clay prior to being put in the microhematocrit centrifuge (UNICO, Dayton, NJ, USA). After the samples were spun down at 10,000 *g* for 6 min at room temperature and the plasma and red blood cells were separated, the capillary tubes were placed on a microhematocrit reader (Jorgensen Laboratories Inc., Loveland, CO, USA) and hematocrit level was determined in duplicate by two trained observers. Body weight was recorded on the same days as blood samples were taken including d 0 of experiment. 

For Experiment 2, blood samples were taken on d 0 (before initial injection), 7, 14 (before additional injection), 21, and 25 (weaning) of experiment for determination of hematocrit levels. The hematocrit level was determined using the same procedures used in Experiment 1. Body weight was recorded on the same days as blood samples were taken.

### 2.4. Statistical Analysis

Growth performance and blood data were analyzed by ANOVA for a randomized complete block design blocked by litter, sex, and body weight using PROC MIXED of SAS (version 9.2; SAS Inst. Inc., Cary, NC, USA) in both Experiment 1 and 2. Models included the treatment as a fixed effect and the sex within litter, litter, and litter × treatment as random effects. The experimental unit was the individual pig. Outlier analysis within each treatment and day was performed using the Grubb’s test outlier calculator (GraphPad Software, https://www.graphpad.com/quickcalcs/grubbs1/, accessed on 26 October 2022, San Diego, CA, USA) and no outlier was identified. There was no piglet death throughout the entire experimental period in both experiments. Least squares means were separated using the PDIFF option of SAS. Statistical differences were considered significant at *p* < 0.05 and tendency at *p* < 0.10.

## 3. Results

In Experiment 1, there was no significant difference in growth performance among the treatments (Table 1).

For hematocrit level, there was no difference in hematocrit levels among treatments at d 15 of experiment (Table 2). The Iron100 and Iron200 treatments had greater hematocrit levels than the Control treatment at d 22 and 26 of experiment (*p* < 0.05). However, the hematocrit level was greater in the Iron200 treatment than the Iron100 treatment at d 26 of experiment (*p* < 0.05). Regarding changes in hematocrit level, the Iron100 and Iron200 treatments had greater increases than the Control treatment in d 15 to 22 and d 15 to 26 of experiment (*p* < 0.05) with no difference between the Iron100 and Iron200 treatments. In d 22 to 26 of experiment, the Iron200 treatment tended to have a greater increase in hematocrit level than the Iron100 treatment (*p* = 0.09).

In Experiment 2, there was no significant difference in growth performance among the treatments (Table 3) similar as Experiment 1.

For hematocrit level, the Iron200b treatment had greater hematocrit levels than the Iron100b treatment at d 14 (*p* < 0.05), 21 (*p* = 0.06; tendency), and 25 (*p* < 0.05) of experiment (Table 4). The changes in hematocrit levels tended to be greater for the Iron200b treatment than the Iron100b treatment in d 7 to 14 (*p* = 0.08) and 0 to 14 (*p* = 0.06) of experiment. With the additional injection (at d 14 after the first injection), the Iron100b treatment had greater increases in hematocrit levels than the Iron200b treatment for d 14 to 21 and 14 to 25 (*p* < 0.05) of experiment.

## 4. Discussion

In pig production, it is a common practice to inject newborn piglets with iron to enhance hematological status in the body as newborn piglets are susceptible to iron deficiency anemia [1,2]. Previous studies demonstrated that 150 to 200 mg of iron injection at birth was sufficient for preventing piglets from anemia during 21 d of suckling period but a single iron injection may not be adequate to prevent anemia past 21 d post-partum [5,14,17]. The suckling piglet grows fast and have approximately 4 times greater body weight at weaning compared with at birth, resulting in greater susceptibility to iron-deficiency anemia at weaning [7,12,16]. As increased hematocrit and hemoglobin levels could result in increased postweaning growth rate [16], an additional iron supplementation may be necessary when the pigs are weaned over 21 d of age to maintain high hematocrit and hemoglobin levels at weaning. In order to supplement iron to piglets, various iron administration routes such as in drinking water, via creep feeding and oral paste, etc. have been used for suckling piglets [9,10,18]. However, due to low absorption rate of iron supplemented orally, the intramuscular injection may be the most efficient way to ensure that the sufficient iron is provided to piglets to avoid iron deficiency anemia [10,11]. Therefore, the current study evaluated the effect of additional iron injection at d 15 to 16 post-partum (11 d before weaning) on preweaning growth performance and hematocrit status of piglets weaned around d 28 of age.

There was no effect of additional iron injection prior to weaning on preweaning growth performance in Experiments 1 and 2. This result agrees with previous studies [13,14,19]. Bruininx et al. [19] reported that double intramuscular injection of 200 mg of iron to piglets at d 3 and 21 of age did not increase growth rate until weaning (d 28 of age) compared with a 200 mg of single iron injection at d 3 of age. Williams et al. [14] also reported that an additional 100 mg of iron injection to piglets 10 d before weaning (d 21 of age) after a 200 mg of iron injection at d 3 of age had no effect on preweaning growth performance compared with a 200 mg of single iron injection at d 3 of age. Chevalier [13] reported that when piglets were injected with a 150 mg of additional iron 4 d before weaning (18 to 24 d of age) after a 150 mg of iron injection at d 1 of age, there was no effect on preweaning growth rate. Although, the additional iron injection to piglets prior to weaning did not impact preweaning growth performance in the current study, further studies are necessary to reach the conclusion on preweaning growth performance due to insufficient number of replicates used in the current study. The suckling piglet has high daily iron requirement at 7 to 16 mg per d during the suckling period with a fast growth rate over 250 g per d [1,2]. However, the sow milk contains 0.2–4 mg iron per L with the 60–90% absorption rate, which result in approximately 1 mg iron/d absorbed by the piglets [1,20]. This indicates that the piglets could not meet their daily iron requirement over the suckling period and need an iron injection at birth. Furthermore, daily gain during suckling period increased from the first week to weaning [21] and hemoglobin and hematocrit decreased from d 17 to 21 of age after the iron injection at birth [6,17]. This supports the suggestion that an additional iron injection may be needed when they are weaned at older age over d 21 of age. Despite this the current results contradict this as no improvement in preweaning growth rate of pigs weaned around 27 d of age was observed. The effect of additional iron supplementation to piglets before weaning may depend on the administration routes. Previously, Yu et al. [9] suggested that creep feeding with 175 mg Fe/kg diet from d 7 of age might contribute the certain amount of iron to the piglets for sufficient overall growth. However, Loh et al. [18] reported that a 200 mg of iron supplementation to piglets at birth in a paste form and via drinking water (32 mg/mL) resulted in lower growth rate and hemoglobin level in the suckling period weaned at d 28 of age than those supplemented with iron via the intramuscular injection even though the piglets were offered a creep feed containing 1,000 mg/kg of ferrous sulfate from d 7 of age. Although the creep feed, oral paste, drinking water, and milk replacer could provide the certain amount of iron to the piglets, orally supplemented iron needs to be absorbed and its absorption rate depends on the iron status in the body, iron intake, and interaction with other trace minerals such as copper and zinc, other dietary factors such as phytate, and gut closure in newborn piglets [18]. Furthermore, it has been reported that oral supplementation of iron to piglets was not efficient due to low absorption rate caused by low gastric acid secretion, immature digestive tract reducing the absorption and weak digestive tract motility [10,22]. Thus, the iron supplementation via oral administration routes may require multiple of continuous administration of iron [18]. Therefore, an additional iron injection could be an efficient way to supplement pigs with iron as there is no absorption process from the gastrointestinal tract. 

In the postweaning period, Chevalier [13] reported that the 150 mg of additional iron injection to piglets 4 d before weaning enhanced postweaning growth rate in a 27 or 30 d of nursery period. Joliff and Mahan [12] also found that a 200 mg of iron injection to piglets at birth plus 100 mg of iron injection d 10 of age (7 d before weaning) slightly increased growth rate in the early postweaning period (d 0–7 postweaning) compared with a 200 mg of single iron injection at birth although it did not affect preweaning growth performance. In contrast, Williams et al. [14] reported no effect of the additional iron injection to piglets prior to weaning on pre- and post-weaning growth performance in a 42 d of nursery period. The response of piglets to an additional iron injection may depend on the fact that the translation of additionally injected iron to growth may require several days for injected iron to be absorbed, reserved in the storage sites, and then subsequently transported to bone marrow for erythropoiesis that enhances hematological status [17], and this could explain that the increase in hemoglobin and hematocrit levels by iron injection occurred 4 to 6 d after the injection [10,17]. Therefore, an additional iron injection before weaning might not show any performance improvement in pre-weaning period and rather it may improve the growth in early postweaning period [12,13]. In addition, this may indicate that injected iron levels (150 vs. 100 mg) and timing (4 d vs. 10 d before weaning) for the additional iron injection could also affect the effectiveness of the second iron injection on pre- and post-weaning growth performance in pigs. Therefore, further studies are needed to demonstrate the effect of varying levels and timing of additional iron injection on pre- and post-weaning growth performance and hematological parameters. Interestingly, previous studies reported that the effect of iron level injected at birth and second iron injection was shown only in early postweaning period but not maintained until later postweaning and growing-finishing periods [12,14]. It may be associated with dietary iron in nursery and later phase diets, which may be realized after the early postweaning period, and dietary iron supplementation has more influence in postweaning growth than iron injection treatment [12]. Regardless of the number of injection, the injectable iron source could be an important factor that affects the efficacy of iron injection to piglets. Iron dextran and gleptoferron are common forms of iron used in swine production [23]. Although they showed similar results in increasing hemoglobin and hematocrit levels, those sources have different bioavailability and mechanism for uptake and metabolism [23,24]. Sperling et al. [23] reported that gleptoferron injection to piglets at birth (d 1–2 of age) could result in higher hemoglobin and hematocrit levels at d 31 of age than those injected with iron-dextran at birth with approximately 5 times greater plasma iron level that could maintain more sustainable iron supply to the piglets. A similar result was reported by Morales et al. [24] in which gleptoferron had higher bioavailability and absorption of iron than iron dextran. In contrast, Pollmann et al. [25] reported that iron dextran treated pigs tended to have greater hemoglobin levels at d 50 of treatment and hematocrit levels at d 10 of treatment than gleptoferron treated pigs. However, there was no difference in growth performance between iron-dextran and gleptoferron reported by these previous studies [24,25]. Therefore, it is also needed to demonstrate the effect of injectable iron sources for second iron injection on pig performance and hematological status due to the difference in bioavailability and absorption of iron between those products. 

It is also worth to note that there could be a potential effect of litter size and average birth weight of piglets in the response of pigs to the iron injection level. The litter size is negatively associated with average piglet birth weight [26,27] and the greater birth weight results in the greater weaning weight [27]. The average birth weight in the current 2 studies were greater than 1.6 kg due to slightly lower average litter size than in the commercial sow production. Joliff and Mahan [12] reported that heavy (>1.5 kg) and light (<1.5 kg) birth weight pigs injected with 200 or 300 mg of iron at birth responded differently to dietary iron levels of 80 to 240 mg in the nursery diets for their postweaning growth. Although the experiment in Joliff and Mahan [12] was not for the second iron injection, the results indicated that the piglet birth weight and iron injection level at birth could be factors affecting the response of pigs to dietary iron level after weaning. As heavy weaning weight pigs has low hemoglobin and hematocrit levels [12] and a positive relationship between hematocrit level and postweaning average daily gain [16], enhancing hematological status of weaning pigs is important to ensure their postweaning growth and reduce iron-deficient anemia that is more likely to happen with large pigs. 

The current study only measured hematocrit level for hematological status of piglets after birth as an indicator as this study primarily focused on the effect of second iron injection in increasing the content of red blood cells in blood that could potentially be useful to examine postweaning growth along with hemoglobin level [16]. The hematocrit, the volume percentage of red blood cells in blood, is one of the main blood parameters used to measure iron status of pigs as the hematocrit is declined by low iron supply [6]. There is a positive correlation between hematocrit and hemoglobin level [16,28] and Joliff and Mahan [12] reported that because of more pronounced response in hematocrit to the weight of pigs than hemoglobin, the hematocrit may be a better indicator of iron status of young pigs. Therefore, the hematocrit level could explain the hematological status associated with iron injection. However, additional hematological parameters are needed for more comprehensive evaluation for the second iron injection to pigs. Heidbüchel et al. [6] reported that a hematocrit level in the blood of suckling piglets lower than 33 to 34% is considered as an iron deficiency. In Experiment 1 and 2, a 200 mg of iron injection at birth could maintain hematocrit level greater than 33% until the second injection. Without an additional iron injection in Experiment 1, hematocrit levels in the control pigs had a decline close to the anemic level. In addition, a 100 mg of iron injection at birth in Experiment 2 resulted in hematocrit level lower than 33% until the second iron injection. These results indicate that a single iron injection and low level of iron injected at birth are not sufficient to provide enough iron to suckling piglets weaned at older age, and thereby those pigs are likely to be anemic after weaning. The additional iron injection 11 d before weaning increased hematocrit levels at weaning (d 26 to 27 of age) over 37% in both experiments indicating that an additional iron injection before weaning is effective to maintain sufficient iron reserve, hemoglobin and hematocrit levels. It has been reported that the additional iron injection 4 to 10 d prior to weaning increased hematocrit levels at weaning (21 d of age) [13,14], which agrees with the current study results. However, in Experiment 1, the additional injection with 200 mg of iron at d 15 of experiment (11 d before weaning) increased hematocrit level at weaning to be greater than that of 100 mg of iron because there was a further increase of hematocrit level in d 22 to 26 of experiment in the 200 mg iron treatment, which resulted from obviously greater iron level in the additional injection. Although there are several previous researches that demonstrated the effect of additional iron injections on hematological status of suckling piglets, those researches had only one treatment for the second iron injection prior to weaning [12,13,14]. In the current study, the results with varying levels of iron in the second injection indicates that the level of iron injection in a second dose could affect the magnitude of hematocrit change until weaning. 

There are inconsistent results with high level of iron injection to piglets at birth as Joliff and Mahan [12] reported similar or slightly lower weaning weight when the piglets received a 300 mg of iron injection at birth compared with 200 mg of iron injection. However, other previous studies [17,29] reported no difference in growth performance of piglets when the piglets injected with over 300 mg of iron at birth. The excess of iron administered to piglets in a single or double injection could be toxic to the piglets as iron could produce hydroxyl radical that causes oxidative stress and DNA damage [1,10]. The split iron injection that could additionally supply iron to piglets before weaning, could be a possible strategy to both satisfy the iron requirement and reduce toxic responses of pigs to high dose of iron in the suckling period as after the second dose of iron-dextran in the split iron injection protocol, the stimulatory effect of injected iron on hepcidin mRNA expression was reduced [10]. Therefore, the second iron injection could be beneficial to supplement more iron to piglets when the hematological status is reduced in the later stage of suckling period and have greater bioavailability of iron in the second dose. 

In Experiment 2, the hematocrit levels increased by the either 100 or 200 mg of iron injection at birth with a greater increase in the first 14 d after the first iron injection when the 200 mg of iron was injected than 100 mg of iron, which agrees with the previous studies reported [14,17], increasing level of iron injected to newborn piglets increased hematocrit levels linearly during the suckling period. After the 200 mg of iron was additionally injected to all piglets at 14 d of experiment (11 d before weaning) in Experiment 2, there was a greater increase in hematocrit level when the low level of iron (100 mg) was injected at birth compared with the 200 mg of iron at birth as the 100 mg of iron injection at birth resulted in lower hematocrit levels at the time of second iron injection than the 200 mg. The result of the current study indicates that lower hematocrit level at the time of additional iron injection (32.5% vs. 37.4%) means that there may be more room for the increase in hematocrit level by the additional iron injection. Gentry et al. [30] reported that hemoglobin status of pigs at weaning affected growth rate after weaning as pigs with a high hemoglobin status had greater feed intake resulting in higher energy retention that could enhance postweaning growth performance of pigs. Furthermore, Bhattarai et al. [16] reported that there was a positive relationship between hematological status at weaning (hemoglobin, hematocrit, and red blood cell) and postweaning growth rate in which an increase of 1.0 g hemoglobin per dL blood could result in an improvement of 17.2 g of weight gain indicating an importance of hematological status of piglets at weaning in their postweaning growth performance. Therefore, when the low level of iron was injected to piglets at birth, an additional iron injection may be needed for piglets to maintain sufficient iron reserve and normal hematological status at weaning for postweaning growth. It is worth to note that the 200 mg of iron injection at birth still maintained greater hematocrit levels from the day of additional iron injection to weaning compared with the 100 mg of iron injection at birth in Experiment 2, which indicates that the greater level of iron injected to piglets in the suckling period by either single or double injection could result in the greater hematocrit level at weaning [12].

In the current study, 1 to 2 d of age were used to inject iron at birth. Previous studies [8,31] used different days of age for the first iron injection at birth within the first week of life. Although the current study could not demonstrate the effect of first iron injection timing on second iron injection, Williams et al. [8] reported that increasing age of first iron injection at birth had a linear decrease in hemoglobin and hematocrit levels at weaning while there was a marginal improvement in preweaning growth rate when the piglets received iron injection until 4 or 6 after birth. However, Egeli and Framstad [31] reported the greater hemoglobin levels at d 7 of age in pigs injected with iron dextran subcutaneously on d 1 of age compared with those treated on d 3 and 4 of age although there was no difference in hemoglobin level on d 14 and 21 of age. Therefore, the timing of first and second iron injections and the amount of iron injected at birth and second injection have influences in hematological status of piglets and potentially in their postweaning growth performance. 

## 5. Conclusions

The additional iron injection to suckling piglets increased hematocrit levels with a greater value at weaning when the piglets received the higher level of additional iron injection. The additional iron injection increased hematocrit levels greater when the piglets received low level of iron injection at birth than high level while the additional iron injection after the high level of iron injection at birth still maintained greater hematocrit levels at weaning than after the low level of iron injection at birth. Therefore, the additional iron injection before weaning has a potential to improve hematocrit levels at weaning although further studies are needed to demonstrate its effect on production measures with more hematological parameters, especially for postweaning period.

## Figures and Tables

**Table 1:** Preweaning growth performance of pigs injected with additional iron before weaning in Experiment 1 ^1^.

	Treatment ^2^			SEM ^3^	*p*-Value
	Control	Iron100	Iron200		
**Body weight, kg**					
d 0 ^4^	1.68	1.63	1.64	0.10	0.93
d 15 ^5^	5.50	5.47	5.48	0.22	0.99
d 22	7.91	7.82	7.96	0.31	0.92
d 26 ^6^	9.03	9.08	9.34	0.41	0.78
**Average daily gain, kg/d**					
d 15–22	0.345	0.336	0.353	0.025	0.80
d 22–26	0.281	0.315	0.345	0.039	0.52
d 15–26	0.322	0.329	0.350	0.028	0.69

^1^ *n* = 8 per treatment from 4 litters; ^2^ Control: no additional iron injection, Iron100: additional intramuscular injection of 100 mg iron-dextran, and Iron200: additional intramuscular injection of 200 mg iron-dextran; ^3^ SEM, standard error of the mean; ^4^ All piglets were injected with 200 mg of iron-dextran at d 1 of age; ^5^ All piglets were injected additionally with iron-dextran at d 16 of age (11 d before weaning) based on the treatment after blood collection; ^6^ All piglets were weaned at d 27 of age.

**Table 2:** Hematocrit level of pigs injected with additional iron before weaning in Experiment 1 ^1^.

	Treatment ^2^			SEM ^3^	*p*-Value
	Control	Iron100	Iron200		
**Hematocrit, %**					
d 0 ^4^	-	-	-	-	-
d 15 ^5^	35.56	34.44	35.44	0.70	0.27
d 22	33.81 ^b^	38.81 ^a^	39.81 ^a^	1.12	0.01
d 26 ^6^	34.25 ^c^	38.75 ^b^	41.19 ^a^	1.38	0.01
**Change, %**					
d 15–22	−1.75 ^b^	4.38 ^a^	4.38 ^a^	0.78	0.01
d 22–26	0.44 ^ab^	−0.06 ^b^	1.38 ^a^	0.53	0.09
d 15–26	−1.31 ^b^	4.31 ^a^	5.75 ^a^	1.07	0.01

^a–c^ Means within the same row without a common superscript differ (*p* < 0.05); ^1^ *n* = 8 per treatment from 4 litters; ^2^ Control: no additional iron injection, Iron100: additional intramuscular injection of 100 mg iron-dextran, and Iron200: additional intramuscular injection of 200 mg iron-dextran; ^3^ SEM, standard error of the mean; ^4^ All piglets were injected with 200 mg of iron-dextran at d 1 of age; ^5^ All piglets were injected additionally with iron-dextran at d 16 of age (11 d before weaning) based on the treatment after blood collection; ^6^ All piglets were weaned at d 27 of age.

**Table 3:** Preweaning growth performance of pigs injected with different levels of iron at birth and additional iron before weaning in Experiment 2 ^1^.

	Treatment ^2^		SEM ^3^	*p*-Value
	Iron100b	Iron200b		
**Body weight, kg**				
d 0 ^4^	1.59	1.60	0.10	0.90
d 7	3.18	3.21	0.26	0.89
d 14 ^5^	5.04	4.98	0.44	0.82
d 21	7.11	6.98	0.65	0.77
d 25 ^6^	8.22	8.10	0.75	0.79
**Average daily gain, kg/d**				
d 0–7	0.227	0.230	0.023	0.89
d 7–14	0.266	0.254	0.027	0.37
d 14–21	0.295	0.285	0.031	0.73
d 21–25	0.280	0.280	0.031	0.97
d 0–14	0.246	0.242	0.025	0.79
d 14–25	0.289	0.283	0.029	0.74
d 0–25	0.265	0.260	0.026	0.77

^1^ *n* = 10 per treatment from 5 litters; ^2^ Iron100b: intramuscular injection of 100 mg iron-dextran at birth with a 200 mg of additional iron-dextran injection 11 d before weaning and Iron200b: intramuscular injection of 200 mg iron-dextran at birth with a 200 mg of additional iron-dextran injection 11 d before weaning; ^3^ SEM, standard error of the mean; ^4^ All piglets were injected intramuscularly with iron-dextran at d 1 to 2 of age based on the treatment after blood collection; ^5^ All piglets were injected intramuscularly with a 200 mg of iron-dextran at d 15 to 16 of age (11 d before weaning) after blood collection; ^6^ All piglets were weaned at d 26 to 27 of age.

**Table 4:** Hematocrit level of pigs injected with different levels of iron at birth and additional iron before weaning in Experiment 2 ^1^.

	Treatment ^2^		SEM ^3^	*p*-Value
	Iron100b	Iron200b		
**Hematocrit, %**				
d 0 ^4^	26.95	25.45	1.59	0.54
d 7	31.50	33.70	1.06	0.22
d 14 ^5^	32.50	37.40	1.13	0.01
d 21	37.50	40.00	0.96	0.06
d 25 ^6^	37.90	40.75	0.59	0.01
**Change, %**				
d 0–7	4.55	8.25	1.75	0.17
d 7–14	1.00	3.70	0.82	0.08
d 14–21	5.00	2.60	0.61	0.04
d 21–25	0.40	0.75	0.74	0.64
d 0–14	5.55	11.95	2.11	0.06
d 14–25	5.40	3.35	0.74	0.04
d 0–25	10.95	15.30	1.89	0.14

^1^ *n* = 10 per treatment from 5 litters; ^2^ Iron100b: intramuscular injection of 100 mg iron-dextran at birth with a 200 mg of additional iron-dextran injection 11 d before weaning and Iron200b: intramuscular injection of 200 mg iron-dextran at birth with a 200 mg of additional iron-dextran injection 11 d before weaning; ^3^ SEM, standard error of the mean; ^4^ All piglets were injected intramuscularly with iron-dextran at d 1 to 2 of age based on the treatment after blood collection; ^5^ All piglets were injected intramuscularly with a 200 mg of iron-dextran at d 15 to 16 of age (11 d before weaning) after blood collection; ^6^ All piglets were weaned at d 26 to 27 of age.

## Data Availability

All available data are included in the present paper.
